# Obstetric medical emergency teams are a step forward in maternal safety!

**DOI:** 10.4103/0974-2700.70755

**Published:** 2010

**Authors:** Hanan M F Al Kadri

**Affiliations:** Obstetrics & Gynecology, King Saud bin Abdulaziz University for Health Sciences, College of Medicine, King Abdulaziz Medical City, Riyadh, Kingdom of Saudi Arabia

**Keywords:** High risk pregnancy, quick response team, rapid response team

## Abstract

**Background and Aim:**

The medical emergency team (MET) system was introduced successfully worldwide. With the exception of a few research publications, most of the described teams are based on patients’ medical rather than obstetric management. The objective of this study was to review literature on the outcome of obstetric MET implementation.

**Materials and Methods:**

Systematic review has been done through searching MEDLINE, the Cochrane Library, relevant articles references, and contact with experts. The author and one other researcher independently selected literature on the establishment or implementation of obstetric MET. There were no restrictions on language, sample size, type of publication, or duration of follow up.

**Results:**

Three publications were identified: Catanzarite *et al*., Gosman *et al*., and Skupski *et al*. They were heterogeneous in terms of the method of implementation and the outcomes discussed. None of them discussed obstetric MET implementation in developing countries.

**Conclusion:**

In the literature, there is a lack of reporting and probably of implementation of Obstetrics METs. Therefore, there is a need for more standardized experiences and reports on the implementation of various types of Obstetrics METs. We propose here a design for Obstetrics METs to be implemented in developing countries, aiming to reduce maternal mortality and morbidity resulting from obstetric hemorrhage.

## INTRODUCTION

The Medical emergency team (MET) system was introduced to treat patients at risk for suffering adverse hospital events.[1] The survival of those patients is determined by the severity of acute illness at admission,[2] the level and quality of care provided,[3,4] and the time interval until this care is delivered.[5,6] Early recognition and prompt treatment of patients at risk for further deterioration may influence the course of illness and decrease morbidity and mortality rates.[7,8] Delayed activation of such teams was associated with an increased risk of death.[9]

METs have been widely adopted, largely based on several studies using historical controls.[9,10] Introduction of the MET seems to improve outcome of hospitalized patients,[11,12] reduce incidence of postoperative adverse outcomes, postoperative mortality rate, and mean duration of hospital stay.[13] This reduction may fluctuate based on differences in the degree of disease complexity and reversibility between medical and surgical patients.[12] However, despite MET documented benefits, barriers to its activation do exist. These include a fear of criticism, strict adherence to traditional model of activation,[14] and the need for a multidisciplinary, multifaceted education/orientation system for involved clinical staff.[15] In a literature review on MET, Barbeti *et al*.[16] found that the evidence in support of MET, is not consistent. Issues such as education, resources, and communication are vital for successful implementation. Trying to validate the role of METs, Chen found that the implementation of METs has increased the proportion of early emergency team calls, while the rate of cardiac arrests and unexpected deaths has decreased.[1] This inverse relationship provides support for the notion that early review of acutely ill ward patients by an emergency team is desirable.

In England and Wales, the National Confidential Enquiries into Maternal Deaths and the Confidential Enquiries into Stillbirths and Deaths in Infancy have repeatedly highlighted the lack of communication and teamwork within the obstetric and midwifery teams as a leading cause of maternal and perinatal deaths.[17]

Notably, most descriptions in the literature are concerned primarily with patients’ medical management and, excluding a limited number of research publications, are rarely focused on the management of high risk obstetric patients.[18–21]

The World Health Organization (WHO) estimates that obstetric hemorrhage complicates 10.5% of all live births in the world.[21] Furthermore, 28% of all direct maternal deaths are directly attributable to hemorrhage, with massive obstetric hemorrhage being a major risk factor for severe maternal morbidity and mortality.[17,19,22] Region of birth,[23] skilled birth attendant at delivery, and health expenditure are found to be key variables that predict maternal mortality at the national level.[24] Substandard care continues to be a major contributor to pregnancy complication, regardless of the country.

The aim of this article is to systemically review the literature on the outcome of Obstetric MET implementation as a method to improve high risk pregnancy care. We also seek to evaluate the effect of MET implementation on maternal mortality and morbidity.

## MATERIALS AND METHODS

We identified literature by searching MEDLINE (1966 to January 2009) and The Cochrane Library (1990 to January 2009). The search terms included were high risk pregnancy, quick response team, rapid response team, medical emergency team, obstetric crisis team, obstetric and hospital system. We also searched the references cited by the identified papers, searched conference proceedings, and contacted experienced persons in the implementation of Obstetrics METs. Two researchers sought all literature that had been published on the establishment or implementation of obstetric MET. There was no restriction on language, sample size, or duration of follow-up/team audit.

Pregnant patients who received hospital-based obstetric medical care were included. Hence, any literature that was published on the outcome of the implementation of any type of obstetric MET on obstetric patients’ safety, was included in this review. Articles that did not report the implementation or the outcome of obstetrics MET were excluded. In cases of multiple articles reporting on the same implementation experience, the one that was most comprehensive was selected.

### Types of interventions

We searched for all the articles that had focused on the introduction of any type or variety of obstetric MET function and its outcomes.

### Types of outcome measures

We evaluated the effect of obstetrics MET implementation on maternal and neonatal safety. We considered the wide variety of outcomes possibly measured by the different researchers and accepted any type of outcome measure related to maternal or neonatal safety.

### Data extraction and quality assessment

The data for each trial were extracted and then crosschecked independently by the author and one other researcher (see Acknowledgements). Discrepancies were settled by discussion.

### Statistical analysis

Due to the novelty of the discussed topic and the expected heterogeneous research methodology, systematic review rather than meta-analysis was performed. Therefore, no statistic calculations were performed.

## RESULTS

Searching the Cochrane library resulted in 687 hits, while searching the Medline database resulted in 422 hits. Only three papers were suitable for inclusion in the review.[18,19,21] The summary of the three identified articles is presented in Table 1.

**Table 1:** Summary of the trials pooled into the review

Gosman 2008	
Method	Obstetric crisis is called condition O (e.g., acute vaginal bleeding, severe abdominal pain and difficulty documenting fetal heart)
	Medical crisis is called condition C
Intervention	Implementation of obstetric crisis team
	Implementation of education and audit programs
	Team responders were defined and their roles were described
Outcome	From June 2005 till December 2006
	Condition O was identified 67 times
	There was increase in the utilization of condition C from 9 to 21/10,000 obstetric admissions
	There was initial delay in using the new system corrected by re-education
	The majority of condition O events involved threats to fetal well being 47/67 (70%)
	62/67 (93%) delivered during the same admission
	The median time from the initiation to the completion of condition O was 6 minutes; quality improvement identified several problems that were corrected by education and system evaluation
Conclusion	1.5 years of experience with Obstetric MET outlined
	Initial low utilization improved with education
	Difficulty in choosing patient safety outcome measures discussed
	No comparison to pre-implementation statistics
Skupski 2006	
Method	Outcome before and after the introduction of patient safety program outlined
	The aim was improving major obstetric hemorrhage care
Intervention	Multidisciplinary obstetrics patient safety team was introduced
	The team included individuals from the divisions of nursing, obstetric anesthesia, maternal fetal medicine, neonatology, surgery (Trauma Team) and blood bank
	Protocols for early diagnosis, assessment, and management were developed
	Trauma team was kept ready to assist the obstetric team
	For cases with suspected hemorrhage, strict management criteria were established
Outcome	Outcomes during the periods of 2000–2001 and 2002–2005 were compared
	Significant increase in cesarean births (*P* < 0.001), repeat cesarean births (*P* = 0.002), and cases of major obstetric hemorrhage (*P* = 0.02)
	Significant improvement in mortality due to hemorrhage (*P* = 0.036), lowest pH (*P* = 0.004), and lowest temperature (*P* < 0.001)
	No differences in measures of severity of obstetric hemorrhage between the two periods
Conclusion	Major obstetric hemorrhage increased during the study period
	Improved outcomes and fewer maternal deaths after implementing systemic approaches
	Hospital systems for caring for women at risk for major obstetric hemorrhage should be improved
Catanzarite 2007	
Method	Rapid response team concept was developed and implemented
Intervention	The team is activated by any team member
	Activation criteria varied for various fetal and maternal emergency reasons
	The activated team includes Labor and Delivery (LandD) charge nurse, in-house obstetrician, anesthesiologist, OR surgical team, neonatologist, and NICU team
	Team activation was by means of hospital-wide overhead page and by beeper
Outcome	Mean decision to OR transfer time was 4.7 minutes
	Mean OR arrival-to-incision time was 6.3 minutes
	Mean total “decision-to incision” time was 11 minutes
	First 6 months of 2006 audit showed 21 cases with times from team activation to delivery of 10.9±4.0 minutes, with a range of 4–19 minutes
	In only four of these cases, the time to delivery was over 15 minutes
	The program was well received by physicians and L and D staff
Conclusion	The program has reduced transfer time of the patients, mean OR arrival to incision and mean total decision to incision

## DISCUSSION

The previous research report’s summary exposes the need for further assessment of the effect of implementing Obstetric MET on maternal and neonatal mortality and morbidity. This should be done through more standardized implementation methods and unified maternal and neonatal indicators to assess the desired outcomes. The number of research articles published on obstetrics METs is limited. This may reflect the international tendency not to give maternal health problems a priority compared with other health problems. The limited experience in the literature does not justify immediate generalizability despite the positive reported outcomes. However, the discussed obstetric METs as well as MET wide implementation experience may be taken as an example guiding the design of such system rather than as a template to be followed blindly. Overall, the design and implementation should be directed by the resources available, skills and the organizing system.

Gosman *et al*.[21] documented the implementation of an obstetrics rapid response team, called O. They identified the needs to implement such a team, implementation challenges, and possible obstructions. They also described the role of education in improving team function. However, they did not report the outcome of such a team by comparing maternal and fetal mortality and morbidity, before and after this team implementation. Gosman *et al*.[21] included in their design various types of obstetric emergencies, such as cord prolapse, fetal distress, and obstetric hemorrhage. On the contrary, Skupski *et al*.[19] designed their team based on situational analysis following two events of maternal deaths directly related to obstetric hemorrhage. They designed their team to deal with hemorrhage cases based on booked patients with well-monitored and planned deliveries, in addition to those with unexpected hemorrhage. The tertiary level of care and the resources available may not be consistent with those available in developing countries.[25] Despite Skupski’s significant results, the model may not be directly applicable for the majority of setups in developing countries. Catanzarite *et al*.[18] developed a rapid response team that reacted to all types of obstetric emergencies. They focused on measuring the outcome in terms of the response time from decision until arrival in the OR or the time of incision. They did not monitor the effect of the system on improving maternal mortality or morbidity and their indicators were very restricted.

Based on the above three limited experiences on obstetrics MET[18–21] and reflecting on the reviewed literature on MET,[9,11,13,26] we can speculate that the implementation of welldesigned Obstetric MET on obstetrics patients may lead to maternal and neonatal outcome improvement, reduce incidence of fetal, maternal and neonatal morbidity and mortality rate, and reduce health care cost.

Globally, 99% of maternal deaths occur in developing countries with hemorrhage as the leading factor.[27] A total of 55% of the deaths occur in Asia, which is responsible for 61% of the world’s births, while Africa accounts for 40% of deaths and 20% of the world’s births. Developed countries account for only 1% of maternal deaths and 11% of all births.[28] In the Kingdom of Saudi Arabia (KSA), hemorrhage has been identified to be the leading cause for maternal mortality.[29] Taking into consideration that the majority of maternal mortality and morbidity cases resulting from hemorrhage are in developing countries, the lack of establishment of these teams in the developed world is reflective of the resource availability and awareness to the effect of MET on improving patients’ outcomes. However, there is a need for a modified protocol to design and establish an obstetric MET that is suitable for implementation in developing countries where there are fewer resources and a higher number of unbooked high risk patients. Furthermore, there should be more attention to the infrastructure of the health care provision necessary in the developing world prior to the implementation of such system.

In developing countries, postpartum and antepartum hemorrhage, expected and emergency difficult obstetrics procedures should be the main focus of Obstetric M*et al*ert criteria. The team responders and their roles should be planned clearly. Those responders should be experienced obstetricians trained on the medical and surgical management of obstetric hemorrhage, as well as experienced midwives. For proper resource utilization at the time of team alert, a clinical nurse/midwife within the same setup should respond first, assist in patient care, and call on expertise from a centralized hospital or regional pool. This centralization should be designed in a way that guarantees arrival at a central facility within a maximum of 30 minutes.[30] The on-call obstetric consultant will always be a member of the team. Prior to implementation, regular education and orientation sessions should be conducted. Furthermore, structured training for the obstetric member on the management of both obstetric hemorrhage and complicated obstetrics cases is essential. Criteria for training and promotion should be created.

The team should have an access to information about the incidence of the following: maternal mortality and morbidity in the institution and the region, antepartum and postpartum hemorrhage, placenta previa, placenta accreta, and blood transfusion rate and indication. They should have a clear sense of the available resources and how quickly they can be accessed (e.g., blood bank, critical care service, operative rooms, available expertise, etc). These data will be the baseline to be used for program establishment and future program audit.

However, in more advanced setups and in the presence of the required facilities, this team can be expanded to include experienced vascular surgeons, experienced urologists, experienced anesthetists, experienced hematologists, in addition to the experienced obstetrician and experienced nurse/midwife. This will provide additional safety and a high level of management for high risk obstetrics cases. The resources required and the implementation process utilized should be considered carefully.

There is a close relationship between effective teamwork, training and patients’ safety in medicine.[31] The use of high fidelity medical simulations appears to be a promising method for enhancing didactic teamwork training.[32,33] Animal labs with hands on skills may be used for training and privileging the team members. Errors in medicine are most frequently due to an interaction of human factors, like poor teamwork and poor communication rather than individual mistakes; hence, continuous team training is needed. To achieve the desired effect of training in this example, the optimal instructional strategies to improve team performance should be established. This instructional strategy should be combined with rigorous outcomes assessment to diagnose team problems and prescribe targeted solutions.[34]

For proper utilization of the trained team and cost effectiveness, we recommend developing a train-the-trainer program. This will guarantee the presence of skilled physicians trained to manage high risk obstetric patients and will spread the knowledge and training across the region and even across the nation. We believe that the degree of physician and administrative compliance with such a preventive program will be reflected in improved maternal health and safety.[35] Strong physician leadership, a funded administrative core and organized community efforts can be used to develop and sustain an effective obstetrics safety program. Two factors may hamper the program’s progress: the possible serious gaps in evidence for best practice in some settings and the lack of large-scale investment in maternity services in others.[36] Train-the-trainers programs and proper advertisement, on top of funded outreach programs, will play a role in eliminating the negative effect of these factors on the success of this project.

Such a team will require extra resources and manpower. However, with the implementation of train-the-trainers and outreach programs, the resource burden will be minimized and the benefits will reach a wider variety of the population, resulting in patients’ safety. We believe that such a team structure deserves proper standardized implementation and auditing criteria. Its effectiveness in reducing maternal mortality resulting from hemorrhage will be a major contribution to maternal safety.

## CONCLUSION

Obstetrics MET represents an important addition to safety in obstetrics, and may lead to standardized, efficient, and timely care for the pregnant population. In the literature, there is a lack of reporting on the outcome of the implementation of such teams. Particularly in the developing world, there is a need for a wider implementation of such team activities with standardized maternal and neonatal indicators for auditing process. The suggested Obstetric MET may not solve the problem completely, but it would likely make a small, but important impact on the number of patients who will sustain unexpected obstetric hemorrhage without access to skilled health care providers.
